# Regulation of Eosinophil and Group 2 Innate Lymphoid Cell Trafficking in Asthma

**DOI:** 10.3389/fmed.2017.00136

**Published:** 2017-08-11

**Authors:** Marie-Chantal Larose, Anne-Sophie Archambault, Véronique Provost, Michel Laviolette, Nicolas Flamand

**Affiliations:** ^1^Centre de Recherche de l’Institut Universitaire de Cardiologie et de Pneumologie de Québec, Faculté de Médecine, Département de Médecine, Université Laval, Québec City, QC, Canada

**Keywords:** eosinophil, group 2 innate lymphoid cells, 2-arachidonoyl-glycerol, chemokine, eotaxin, asthma

## Abstract

Asthma is an inflammatory disease usually characterized by increased Type 2 cytokines and by an infiltration of eosinophils to the airways. While the production of Type 2 cytokines has been associated with T_H_2 lymphocytes, increasing evidence indicates that group 2 innate lymphoid cells (ILC2) play an important role in the production of the Type 2 cytokines interleukin (IL)-5 and IL-13, which likely amplifies the recruitment of eosinophils from the blood to the airways. In that regard, recent asthma treatments have been focusing on blocking Type 2 cytokines, notably IL-4, IL-5, and IL-13. These treatments mainly result in decreased blood or sputum eosinophil counts as well as decreased asthma symptoms. This supports that therapies blocking eosinophil recruitment and activation are valuable tools in the management of asthma and its severity. Herein, we review the mechanisms involved in eosinophil and ILC2 recruitment to the airways, with an emphasis on eotaxins, other chemokines as well as their receptors. We also discuss the involvement of other chemoattractants, notably the bioactive lipids 5-oxo-eicosatetraenoic acid, prostaglandin D_2_, and 2-arachidonoyl-glycerol. Given that eosinophil biology differs between human and mice, we also highlight and discuss their responsiveness toward the different eosinophil chemoattractants.

## Introduction

Asthma is a respiratory disease characterized by inflammation and hyperresponsiveness of the airways and roughly affects 300 million people worldwide (1). Eosinophils play a pivotal role in asthma by generating many mediators inducing bronchoconstriction and/or contributing to inflammation and remodeling (2). Airway eosinophilia is observed in many subjects with asthma and increases with disease severity and exacerbations (3). The anti-inflammatory treatment of asthma is primarily based on inhaled corticosteroids (4). The dose is adjusted to decrease eosinophil counts in the blood and/or in induced sputum, which results in a reduction of asthma exacerbations. However, the chronic use of corticosteroids is linked with significant systemic side effects even at low doses, and some severe asthmatics remain symptomatic and have high sputum eosinophil counts despite the use of high doses of corticosteroids (5). This stresses the need of developing new therapeutics that could limit both bronchoconstriction and inflammation.

Increased eosinophil numbers are observed in many asthmatics, notably those characterized by a Type 2-like inflammation, characterized by an increased production of the cytokines interleukin (IL)-4, IL-5, and IL-13 (6). As such, it is well accepted that the Type 2 cytokines IL-4, IL-5, and IL-13 are linked to increased eosinophil numbers, either by promoting eosinophil survival (IL-5) or by inducing the production of eosinophil chemoattractants (IL-4 and IL-13) (7, 8). While T_H_2 lymphocytes participate in the release of Type 2 cytokines, group 2 innate lymphoid cells (ILC2) are being increasingly recognized as a significant source of Type 2 cytokines as well (9, 10). Asthma treatments that focused on blocking Type 2 cytokines (IL-4, IL-5, and IL-13) decrease blood or sputum eosinophil counts and asthma symptoms in subjects with severe asthma presenting a high eosinophil count in their induced sputum (11–25). This article reviews the current evidence regarding eosinophil and ILC2 chemoattractants and their involvement in asthma and its severity.

## Discovery Timeline of the Main Eosinophil Chemoattractants

The extensive investigation of how eosinophils were recruited really began in the 1970s. Complement component 5a (C5a) has been known to induce guinea pig eosinophil migration since 1970 (26–29), and its impact on human eosinophils was documented in 1973 (26). Histamine was next documented as an eosinophil chemoattractant in 1975 (30) although its effect is limited (31–34).

In 1980s, other eosinophil chemoattractants were characterized, notably platelet-activating factor (PAF), leukotriene (LT) B_4_, and *N*-formylmethionyl-leucyl-phenylalanine (fMLP). Numerous reports indicate that PAF induces the migration of eosinophils (29, 35–41). Even if LTB_4_ is mainly characterized as a neutrophil chemoattractant, it also induces human eosinophil migration (29, 37, 42, 43). fMLP is a weak chemoattractant for eosinophil migration: some studies unraveled a weak migration of eosinophils (29, 37, 44, 45) while others did not find any effect (38, 46).

The expansion of the chemokine field in the 1990s allowed the characterization of additional eosinophil chemoattractants. CCL5 [regulated on activation, normal T cell expressed and secreted (RANTES)] was the first chemokine documented as a human eosinophil chemoattractant in 1992 (47) and was shown to induce both the migration and transmigration of human eosinophils (48–57). The effect of CCL3 (MIP-1α) on human eosinophil migration was also evaluated in 1992 (47). However, the ability of CCL3 as an eosinophil chemoattractant is low, as later reports indicated that at optimal concentration, the CCL3-induced migration of eosinophil corresponded to about 33% of that induced by CCL5 (48, 52, 57). Of note, one study showed that ~20% of individuals responded to CCL3 to the same extent than CCL11, while the others poorly responded to CCL3 and this was linked to CCR1 (58). In mid-1990s, other chemokines were tested for their ability to elicit human eosinophil migration, notably CCL7 (MCP-3), CCL8 (MCP-2), and CCL13 (MCP-4) (34, 48, 50–53, 55–57, 59, 60). However, their impact on human eosinophil migration was limited.

The discovery of eotaxins was a substantial leap forward in understanding how eosinophils were selectively recruited into the tissues. CCL11 (eotaxin-1) was first discovered by Jose et al. in guinea pigs (61, 62). Two years later it was confirmed as a selective chemoattractant of human eosinophils in 1996 (63) and several studies confirmed its potency in several migration models (55, 64–66). A year later, CCL24 (eotaxin-2) was discovered (67) and was confirmed as being as efficient as CCL11 (34, 55–57, 65). Last but not the least, CCL26 (eotaxin-3) was discovered in 1999 (68, 69), and it is the most efficient eotaxin to induce the migration or transmigration of asthmatic eosinophils (65). Of note, CCL26 appears also critical for eosinophil migration/tissue eosinophilia in other human disorders characterized by eosinophil recruitment, notably eosinophilic esophagitis and Churg–Strauss syndrome (70, 71).

It was also in the mid-1990s that additional bioactive lipids from the 5-lipoxygenase pathway were documented as human eosinophil chemoattractants. 5-Oxo-eicosatetraenoic acid (5-KETE) was identified as a potent chemoattractant of eosinophils in 1996 (72, 73). To this date, 5-KETE is the most efficient human eosinophil chemotactic factor *in cellulo* (41, 43, 65, 66). LTD_4_ was the first cysteinyl leukotriene (CysLTs) to be defined as a direct chemoattractant of human eosinophils (74) but induces a weak migration (75–78). It was also reported that LTC_4_ and LTE_4_ induce an eosinophil migration comparable to LTD_4_ (79).

The new millennia also expanded our knowledge on how human eosinophils could be recruited into the tissue. In that regard, CXCL12 (SDF-1) was shown to induce the recruitment of eosinophils (65, 80, 81). Furthermore, a 2001 study demonstrated that prostaglandin (PG) D_2_ selectively induced the migration of eosinophils, Th2 lymphocytes cells, and basophils (82), and increasing evidence support the development of DP_2_/CRTH2 antagonists for the management of asthma (83). However, PGD_2_ seems to induce a limited recruitment of eosinophils (66, 84–88). Of note, PGD_2_ increases CCL11- and 5-KETE-induced-eosinophil migration (87). Finally, in 2004, the endocannabinoid 2-arachidonoyl-glycerol (2-AG) was identified as an eosinophil chemoattractant (89); this effect of 2-AG involves the CB_2_ receptor and is largely potentiated by IL-3, IL-5, and GM-CSF (66, 90, 91).

## Human Eosinophil Recruitment and Asthma

**Figure 1:**
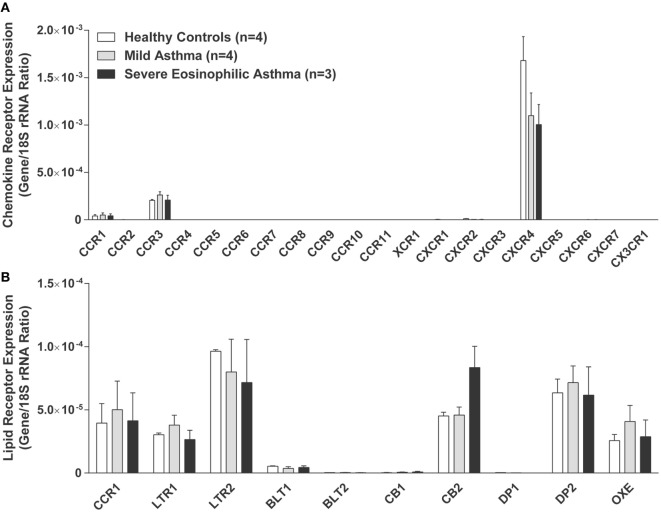
Expression of chemokines and lipid mediator receptors by human eosinophils. Human eosinophils were isolated from the blood of healthy controls, mild asthmatics, and severe eosinophilic asthmatics as defined and described in Ref. (92). mRNAs were quantitated by qPCR array using a custom qPCR array (RT^2^ Profiler qPCR Multiplex Array Kit, Qiagen, ON, Canada). Chemokine receptor expression **(A)** and bioactive lipid receptor expression **(B)** are represented by the ratio between mRNAs and 18S rRNA control. Results are the mean (±SEM) of 3–4 donors for each group. Approval from the local ethics committee was obtained, and all volunteers signed an informed consent form.

As underscored in the previous section, many soluble mediators and chemokines can induce human eosinophil recruitment and thus participate in asthma pathogenesis. In this section, we review how these chemoattractants contribute to eosinophil recruitment in a context of asthma. A differential eosinophil recruitment could be observed in asthma severity and/or during asthma exacerbations if there is a dysregulation in the release of the different chemoattractants or their receptors, notably by desensitization or internalization. To this end, our data (Figure 1) indicate that with the exception of the CXCR4 and the CB_2_ receptors, the expression of chemoattractant receptors do not change, at the mRNA level, in human eosinophils isolated from the blood of healthy subjects, mild and severe eosinophilic asthmatics, as defined in Ref. (92). This supports the notion that perhaps the increased recruitment of eosinophils is rather the consequence of increased chemoattractants in the bronchial tissue.

## Chemokines

The most studied chemokines in asthma are CCL5 and eotaxins, probably because their levels are usually increased in asthmatics compared to healthy controls in all body fluids tested, namely bronchoalveolar lavages (BAL), induced sputum, blood, and bronchial biopsies (92–115). Moreover, these chemokines are linked to poor asthma control and increased eosinophil recruitment to the airways. Indeed, CCL5 levels are greater in induced sputum from poorly controlled asthmatics than from controlled asthmatics (116, 117); subjects undergoing acute exacerbations have higher CCL11 levels in induced sputum and plasma samples than subjects with stable asthma or healthy controls (111, 118–120); and CCL24 and CCL26 expression in airway epithelial cells are associated with lower forced expiratory volume in 1 s (FEV_1_), more asthma exacerbations, and increased sputum eosinophil counts (92, 121). It is not clear whether one chemokine is more important than the others and if we could target these chemotactic proteins to limit eosinophil recruitment and asthma exacerbation. In that regard, different studies evaluated the expression of these chemokines during allergen challenges, and the obtained data rather indicate that eosinophil-recruiting chemokines are not necessarily present at the same time and might have different as well as overlapping roles. CCL5 levels correlate with eosinophil counts in BAL 4 h after the challenge (122), but not 24 h after the challenge (123). CCL11 levels are increased in BAL, induced sputum and bronchial biopsies of asthmatics, and are associated with eosinophil numbers 4 and 24 h after the challenge (104, 124, 125). That being said, one study reported that CCL11 levels are similar in bronchial biopsies from asthmatics before and 24 h after allergen challenge (103). CCL24 expression is significantly increased in bronchial mucosa from asthmatics 48 h after allergen challenge (126), but is similar before and 24 h after allergen challenge (103). As for CCL26, its expression in bronchial biopsies increases 24 and 48 h after allergen challenge (103, 126), but its expression in bronchial submucosa did not correlate with eosinophil counts 48 h after allergen challenge (126). Additionally, some research groups documented the impact of these chemokines on eosinophil migration in asthma *in cellulo*. CCL11 and CCL26 induce a greater migration of eosinophils from asthmatics than from healthy subjects (65, 127). Finally, while most evidence reflects an important role of CCL5 and the eotaxins in asthma, some studies reported that there was no increase in CCL5 or eotaxin expression in BAL, airway epithelium brushings, or bronchial biopsies between asthmatics and healthy controls (92, 103, 121, 128, 129).

Studies on CCL3, CCL7, CCL8, CCL13, and CXCL12 in relation with asthma are limited. Among the latter, CCL13 is better associated with eosinophils and asthma. Its expression is higher in BAL, bronchial biopsies, induced sputum, and plasma samples from asthmatics than from healthy controls (99, 100, 105, 130, 131). One study reported increased CCL3 levels in BAL from asthmatics compared to healthy controls (93). Increased CCL7 levels and CCL7-expressing cells are found in bronchial biopsies and BAL from asthmatics compared to healthy controls (94, 95, 100), and serum CCL8 levels are higher in asthmatics compared to healthy controls (132). CXCL12 levels in bronchial mucosa and BAL are greater in asthmatics than in healthy controls (133, 134), and CXCL12 levels in BAL correlate with eosinophil numbers (134).

## Lipid Mediators and Others

Other soluble mediators might also participate in the recruitment of eosinophils in asthma. In that regard, CysLT_1_ receptor blockade usually decreases eosinophil counts, although it is not clear whether this is a direct or indirect effect (135–144). LTB_4_, histamine, C5a, and PGD_2_ are all associated with asthma, but their involvement in eosinophil recruitment in asthma is not well defined. Even if LTB_4_ levels in blood and exhaled breath condensate are increased in asthma (145–147), the LTB_4_ receptor antagonist, LY293111, decreases neutrophil but not eosinophil counts in BAL from asthmatics (148). As for PGD_2_, some studies demonstrated similar PGD_2_ levels in BAL or induced sputum of asthmatics, atopics, and healthy subjects (149–152), but its levels can increase in the BAL after an allergen challenge (149, 153, 154). Of note, the antagonism of the PGD_2_ receptor 2 (DP_2_/CRTH2) improves lung function and the quality of life of asthmatics compared to placebo (155, 156). Finally, C5a levels are increased in BAL and in induced sputum from asthmatics compared to healthy controls after an allergen challenge (157, 158), and a haplotype of the C5a gene was identified to be protective against asthma (159).

As for PAF, 5-KETE, fMLP, and 2-AG, their association with asthma is not well documented and this requires further investigations. For example, we have no idea to which extent 2-AG and 5-KETE levels are modulated in asthma and its severity.

## Asthma Severity

As underscored with the data from the allergen challenges presented in the previous section, it is not possible to pinpoint one chemoattractant explaining the recruitment of human eosinophils. They rather indicate that they collaborate together and that they might be involved at different times during the asthmatic response. In addition, it is possible that the mediators responsible for eosinophil recruitment might also change as the disease worsens. For example, CCL11 and/or CCL26 levels are greater in induced sputum from severe or moderate asthmatics than from mild asthmatics or healthy controls (92, 160). In plasma samples, CCL11 levels are associated with asthma severity and are not significantly affected by corticosteroid treatment (161). Coleman et al. demonstrated that CCL24 and CCL26, but not CCL11, mRNA expression in bronchial epithelium increases with asthma severity and is associated with sputum eosinophil counts, lower FEV_1_, and more asthma exacerbations (121). In contrast, subjects with severe eosinophilic asthma have lower CCL24 levels in bronchoalveolar lavage fluids and similar CCL24 levels in bronchial epithelial cells compared to healthy controls (92, 121). For CCL5, Saad-El-Din demonstrated that serum CCL5 levels are greater in subjects with severe or moderate asthma as compared to subjects with mild asthma and are associated with blood eosinophil number (114). As for CXCL12, it induces a greater migration of corticosteroid-treated eosinophils than untreated eosinophils and that the expression of the CXCL12 receptor, CXCR4, increases in corticosteroid-treated eosinophils (80), raising the possibility that CXCL12 plays a more important role in unstable severe eosinophilic asthmatics which are taking large doses of corticosteroids.

In asthma, CysLTs levels in induced sputum are increased in moderate asthmatics compared to severe asthmatics and healthy controls (162). Also, similar sputum CysLTs levels were found in severe eosinophilic and non-eosinophilic asthmatics (162). In contrast, exhaled breath condensate levels of CysLTs correlate with asthma severity (163). In mild-to-moderate asthmatics or eosinophilic asthmatics, the CysLT1 antagonist montelukast, alone or in combination with corticosteroids, decreases sputum or blood eosinophil counts (136, 138, 141, 164). On the other hand, severe eosinophilic asthmatics, severe non-eosinophilic asthmatics, and moderate uncontrolled asthmatics have similar sputum or blood eosinophil counts between montelukast-treated and placebo-treated individuals or between montelukast/corticosteroid-treated and corticosteroid-treated asthmatics (165–167). Of note, PGD_2_ and DP_2_/CRTH2 levels are increased in asthma severity in BAL (151, 152), and the DP_2_/CRTH2 antagonist OC000459 improves FEV_1_ and the quality of life of subjects with eosinophilic uncontrolled asthma and steroid-free subjects with moderate persistent asthma (155, 156). Finally, C5a receptor expression on bronchial epithelium is greater in subjects with fatal asthma than mild asthmatics and healthy controls (168).

## Of Mice and Men

The potential and/or documented roles of multiple chemoattractant involved in eosinophil recruitment in asthma underscore the need to revisit this concept and to establish when and how those actors are involved. The development of experimental asthma models with mice, rats, or guinea pigs has been very helpful to broaden our knowledge about asthma pathogenesis and to identify some eosinophil and ILC2 chemoattractants in allergic asthma. However, eosinophils and their functional responses are very different between species (169). In that regard, some chemoattractants and their receptors in humans are not expressed in mice. For instance, the 5-KETE receptor OXE is not expressed in mice (170, 171), resulting in an absence of 5-KETE-induced eosinophil migration (170). Additionally, CCL26 is not expressed in mice (170) and human CCL26 does not induce the migration of mouse eosinophils (172, 173). Furthermore, CCL5 does not induce the migration of mouse eosinophils (172, 174–176). Globally, three of the most efficient human eosinophil chemoattractants described so far (CCL5, CCL26, and 5-KETE) do not induce the migration of eosinophils from mice, illustrating major differences in eosinophil recruitment between mice and humans and underscoring that transposing eosinophil recruitment data from mice to humans might be hazardous. The impact of the different chemoattractants on the migration of eosinophils from humans and mice is summarized in Table 1 in which the number of migrated eosinophils in different migration assays is compared. It should be kept in mind that the presented data involve different eosinophil migration assays and that a true comparison between the presented chemoattractant is somewhat subjective. This is why we defined the different efficiencies using %migration intervals.

**Table 1:** Eosinophil chemoattractants and their receptors of human and mice.

Eosinophil chemoattractants	Human		Mice	
	Receptors	Efficiency	Receptors	Efficiency
CCL11/eotaxin-1	CCR3 (177–179)	++ (55, 56, 64, 66)	CCR3 (172, 180)	++ (181)
CCL24/eotaxin-2	CCR3 (179, 182)	++ (55, 56)	CCR3 (172, 180)	+ (172, 173)
CCL26/eotaxin-3	CCR3 (68, 69)	+++ (65, 68, 69)	CCR3 (172, 180)	− (172, 173)
CCL5/RANTES	CCR1, CCR3 (58, 177, 178, 183, 184)	++ (47, 52, 55, 56)	CCR1, CCR3, CCR5 (172, 180)	− (172, 174, 175)
PAF	PAFR (185, 186)	++ (29, 37, 39, 41)	PAFR (187)	+ (181)
C5a	C5aR (188–190)	++ (29, 47, 52)	C5aR (191, 192)	++ (174, 193)
2-AG	CB_2_ (89, 194)	+ (66, 90)	n/a	n/a
5-KETE	OXE (171, 195, 196)	+++ (41, 43, 66)	n/e	− (170)
LTB_4_	BLT_1_ (197, 198)	+ (29, 37, 64)	BLT_1_ (197)	+ (199)
PGD_2_	DP_2_/CRTH2 (82, 87)	+ (87)	DP_2_/CRTH2 (200, 201)	+ (202, 203)
fMLP	FPR (204–206)	+ (29, 37, 52)	n/a	+ (193, 207)
CCL3/MIP-1α	CCR1, CCR3 (58, 177, 178, 183, 184)	± (47, 48, 52, 57)	CCR1, CCR3 (172, 180)	± (172, 173, 181, 208)
CCL7/MCP-3	CCR1-CCR3 (178, 183, 209)	+ (52, 55)	CCR1–CCR3 (172, 180)	n/a
CCL8/MCP-2	CCR1–CCR3 (183, 184, 209)	+ (52)	CCR1–CCR3 (172, 180)	n/a
CCL13/MCP-4	CCR1–CCR3 (177, 183, 209)	+ (56)	CCR1–CCR3 (172, 180)	n/a
CXCL12/SDF-1	CXCR4 (80, 210)	++ (65, 80)	CXCR4 (172)	n/a
LTD_4_	CysLT_1_, CysLT_E_? (211, 212)	+ (74–77)	CysLT_1_, CysLT_E_? (213)	− (199)

*−: no migration, ±: weak or no migration, +: migration usually between 10 and 30%*.

*++: migration usually between 30 and 50%, +++: migration over 50%*.

*2-AG, 2-arachidonoyl-glycerol; fMLP, N-formylmethionyl-leucyl-phenylalanine; n/a, not available; n/e, not expressed; PFA, platelet-activating factor; PG, prostaglandin*.

## Mediators Promoting ILC2 Recruitment

First identified in 2010, ILC2 are defined as lymphoid cells lacking specific lymphocytes lineage markers and the expression of the DP_2_/CRTH2 and ST2, the IL-33 receptor (214–218). They produce, in response to IL-25, IL-33 or thymic stromal lymphopoietin (TSLP), large amounts of the T_H_2 cytokines IL-5, IL-13 and, to a lesser extent, IL-4. Of note, the number of ILC2 correlate with sputum eosinophils in allergic asthma (219). This suggests that ILC2 might play an important role in asthma (220, 221), especially by directly or indirectly modulating eosinophil survival/recruitment. However, the cellular mechanisms by which ILC2 are recruited to the lungs remain poorly defined and few studies addressed the impact of chemokines or bioactive lipids on the migration of ILC2.

Since IL-25, IL-33, and TSLP are potent activators of ILC2, their ability to induce the migration of ILC2 was first evaluated. IL-33 and TSLP induce a weak migration of human ILC2 (218, 222, 223). However, the impact of IL-25 remains a matter of debate, as one study reported a weak IL-25-induced ILC2 migration (223), while another found no effect of IL-25 (218). PGD_2_ and CysLTs are defined as potent chemoattractants of ILC2. Indeed, PGD_2_ is almost five times more potent than IL-33 (218, 224), and the PGD_2_-induced migration is greater in ILC2 from allergic subjects compared to healthy subjects (224). Furthermore, mice lacking DP_2_/CRTH2 or treated with a DP_2_/CRTH2 antagonist have lower ILC2 levels in the lungs after intranasal administration of PGD_2_ (225). As for CysLTs, ILC2 express the receptor CysLTR_1_ and its expression is increased in atopic subjects (223, 226, 227). Interestingly, a research group recently demonstrated that all CysLTs induce the migration of human ILC2 *in vitro*, LTE_4_ ≫ LTD_4_ > LTC_4_ ≈ IL-33, indicating that perhaps another CysLT receptor might be involved in this process (223).

Although only IL-33, TSLP, PGD_2_, and the CysLTs have been identified as chemoattractants of ILC2, some studies reported that human ILC2 express the chemokine receptor CCR4 and mouse ILC2 express the LTB_4_ receptor BLT_1_ (222, 227). Furthermore, TGF-β increases the basal migration of murine ILC2, which suggests that it could enhance their response to other chemoattractants (228). Other studies are thus needed to delineate how ILC2 migrate to the bronchial tissue.

## Concluding Remarks and Future Directions

This review highlights that many chemokines and soluble mediators are very good to excellent at inducing the migration of eosinophils *ex vivo* and their recruitment *in vivo*. This underscores that targeting eosinophil recruitment as a therapeutic approach in asthma might not be readily successful, as suggested with the attempt at blocking the eotaxin receptor CCR3 (229). Additionally, many questions remain unanswered. For instance, it remains unclear when all those chemoattractants actually play a role during the asthmatic response and this needs to be addressed, notably by defining the presence of all eosinophil and ILC2 chemoattractants in the same samples and at different stages of the disease/exacerbation. Experimental restrictions such as specie (mouse vs. humans) or the number of chemoattractants being investigated in a given study make the obtained data a little blurry, sometimes raising more questions than answering them. In addition, the involvement of the different chemoattractants as the disease worsens remains anecdotal. Given that severe asthmatics are frequently older than mild and moderate asthmatics, it is possible that the set of chemoattractant changes with age and perhaps, with gender as well [keeping in mind that aging modulates sex hormones, which could affect the synthesis of the different chemoattractants as it is the case for 5-lipoxygenase derivatives (230)]. Another important aspect of this review is the illustration that some of the best chemoattractants for human eosinophils are not present or are effectless in murine models (Table 1), raising the question that perhaps data obtained from animal models should be taken cautiously until they are validated in humans. Finally, if ILC2 play a prominent role in asthma as it is proposed from mouse data, it will be of crucial importance to rapidly understand the regulation of their recruitment into the airways, by defining which chemokines, lipids, and other chemoattractants are promoting their recruitment both in mice and humans, as well as all the receptors involved in that process.

## Author Contributions

All the authors listed have made a substantial, direct, and intellectual contribution to the work and approved it for publication.

## Conflict of Interest Statement

The authors declare that the research was conducted in the absence of any commercial or financial relationships that could be construed as a potential conflict of interest.
